# Topography‐Directed Hot‐Water Super‐Repellent Surfaces

**DOI:** 10.1002/advs.201900798

**Published:** 2019-07-30

**Authors:** Pingan Zhu, Rifei Chen, Liqiu Wang

**Affiliations:** ^1^ Department of Mechanical Engineering The University of Hong Kong Hong Kong China; ^2^ HKU‐Zhejiang Institute of Research and Innovation (HKU‐ZIRI) Hangzhou 311300 Zhejiang China; ^3^ Department of Materials Science and Engineering Southern University of Science and Technology Shenzhen 518055 China

**Keywords:** heat transfer, liquid repellency, structure–property relationship, wetting dynamics

## Abstract

Natural and artificial super‐repellent surfaces are frequently textured with pillar‐based discrete structures rather than hole‐based continuous ones because the former exhibits lower adhesion from the reduced length of the three‐phase contact line. Counterintuitively, here, the unusual topographic effects are discovered on hot‐water super‐repellency where the continuous microcavity surface outperforms the discrete microneedle/micropillar surface. This anomaly arises from the different dependencies of liquid‐repellency stability on the surface structure and water temperature in the two topographies. The unexpected wetting dynamics are interpreted by determining timescales for droplet evaporation, vapor condensation, and droplet bouncing. The associated heat transfer process is unique to the wetting states and remarkably distinct from each other in the two topographies. It is envisioned that hot‐water super‐repellent microcavity surfaces will be advantageous for a variety of applications, especially when both self‐cleaning and thermal insulation are imperative, such as clothing for scald protection and digital microfluidics for exothermic reactions.

Repelling hot liquids is relevant to many industrial processes, including heat exchangers,^1^ fuel‐spray impingent,^2^ water desalination,^3^ and additive manufacturing,^4^ as well as to our daily life, such as for scalding protection clothes^5^ and anti‐fouling kitchenware. In these situations, the heat transfer between hot liquids and cold surfaces is affected by the solid–liquid interaction,^6^ which in turn influences the system performance. For example, the heat transfer efficiency determines the vapor flux of distillation in water harvesting;^3^ protective clothes require thermal insulation between hot liquids and human bodies to avoid scalding. Despite its importance, the effort to study the mechanisms of super‐repellency to hot liquids appears very limited.

In contrast to room‐temperature water, hot water complicates the dynamics of liquid wetting and heat transfer with phase change processes, including the evaporation of hot water droplets and condensation of vapor on cold surfaces. Previous studies have mainly been focused on the fabrication of hot‐water super‐repellent surfaces, such as by spray coating, deposition, and electrophoresis‐assisted coating, and their applications for self‐cleaning and oil/water separation,^7^, ^8^, ^9^, ^10^, ^11^, ^12^, ^13^, ^14^, ^15^ whereas only a few studies have reported on wetting mechanisms by hot water.^16^, ^17^ More recently, it was unveiled that hot‐water droplet bouncing is dramatically affected by the size of surface structure;^18^ both nanometric (≈100 nm) and microscale (≈10 µm) features are favorable to hot‐water repellency, while the structure with a dimension in between (≈1 µm) fails at repelling hot water. A cold solid surface can lose the repellency to hot water^5^, ^16^ by three mechanisms: reduced surface tension of hot water,^19^ destruction of surface structures by elevated temperatures (for example, plant leaves with wax coatings^5^, ^17^), and condensation of water vapor that forms liquid bridges connecting the hot water and surface asperities.^16^ To maintain hot‐water super‐repellency, the surface design must overcome all three failure mechanisms. Among them, structure destruction can be easily circumvented by using materials with high melting points. Nevertheless, avoiding failure from the reduced surface tension of hot water and vapor condensation requires a comprehensive understanding of solid–liquid interactions involving temperature differences. Therefore, investigating the role of surface topography on hot‐water wetting is crucial to the design of robust surfaces that are super‐repellent to hot water.

Designs of hot‐water super‐repellent surfaces can be borrowed from superhydrophobic surfaces. Generally, a solid surface is physically roughed with reduced solid–liquid contact areas and chemically modified with low surface energy, on which water droplets are suspended in the well‐known Cassie state^20^ with entrapped air cushions. Surface topography affects the dynamics of liquid wetting. Most superhydrophobic surfaces are composed of discrete architectures with solid extrusions, such as pillars, needles, particles,^21^ and those occurring on lotus leaves,^22^ rather than continuous topologies with interconnected top surface structures including holes,^23^, ^24^ cages,^25^ and those decorating the cuticles of springtails.^26^ The reason for this trend may be that compared to continuous topographic surfaces, discrete surfaces are lower in adhesion due to the reduced length of the three‐phase contact line^27^ and easier to fabricate using well‐developed techniques such as etching and lithography. Considering that continuous and discrete structures are topologically complementary to each other (for example, holes and pillars), systematically comparing their performance on liquid wetting would deepen our fundamental understanding of hot‐water repellency. As such, the present work aims to identify the difference between continuous and discrete surface topographies in repelling hot water by combining experimental studies with theoretical modeling.

Contrary to the common case in superhydrophobic surfaces, here we demonstrate that the continuous topographic microcavities, characterized by the re‐entrant structure and higher breakthrough pressure, outperform the discrete topographic microneedles in repelling hot water for higher static contact angles, lower contact angle hysteresis, and easier droplet rebounding. We interpret their differences in wetting dynamics by the relative magnitudes of the timescales for hot‐water droplet evaporation, vapor condensation, and contact between bouncing droplets and cold surfaces. It is found that heat transfer is related to the wetting states for the two surface topographies, where the entrapped air provides a more effective thermal insulating layer for nonwetted microcavities than wetted microneedles. The hot‐water super‐repellent microcavity surfaces would be useful in a range of applications, including scald‐protection clothing requiring thermal insulation and digital microfluidics for manipulating aqueous droplets with varied temperatures and performing exothermic reactions.

Robust super‐repellent surfaces have to meet three requirements: high apparent contact angles θ* (>150°), low contact angle hysteresis (sliding angle θ_s_ < 10°), and high breakthrough pressure *P*_break_ for the stable Cassie state. Condensation inevitably occurs during the contact between the hot water and cold solid surfaces. Large asperities are designed to provide enough room for accommodating the water condensation to prevent the filling of voids between solid structures. To understand the mechanisms underlying hot‐water super‐repellency, we investigate the wetting and heat transfer processes using microcavity and microneedle architectures (**Figure** **1**A–F), which represent the continuous and discrete surface topographies, respectively.

**Figure 1:**
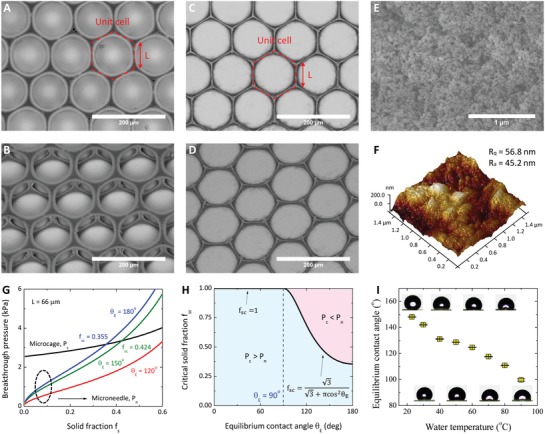
Design and fabrication of the microcavity and microneedle surfaces. A–D) Scanning electronic microscopy (SEM) images of nanoparticle‐coated microcavity surfaces in the top view (A) and 30° tilted view (B) and nanoparticle‐coated microneedle surfaces in the top view (C) and 30° tilted view (D). *L* represents the side length of a hexagonal unit cell (*L* = 66 µm). E) SEM image of nanoparticle coatings. F) Atomic force microscopy (AFM) image of the 3D morphology of nanoparticle coatings. G) Breakthrough pressure for the microcavity (*P*_c_) and microneedle (*P*_n_) surfaces when *L* = 66 µm and θ_E_ = 120°, 150°, and 180°. H) Phase map for *P*_c_/*P*_n_ with respect to the critical solid fraction *f*_sc_ and equilibrium contact angle θ_E_. I) Variations in the equilibrium contact angle θ_E_ with the temperature of water droplets on the nanoparticle‐coated surfaces.

Microcavities (Figure 1A,B and Figure S1A,B, Supporting Information) are fabricated by droplet microfluidics with precisely controlled morphologies.^28^, ^29^ Owing to the re‐entrant structures (the minimum geometrical angle ϕ_min_ = 0°), the microcavities exhibit robust omniphobicity in the air^23^ and under‐liquid super‐repellency in virtually any two‐fluid systems.^25^ Peeling off the top surface of microcavities produces microneedle structures resembling micropillars (Figure 1C,D and Figure S1C,D, Supporting Information). Both of the microstructures are coated with silanized silica nanoparticles dispersed in isopropanol (Figure 1E, nanoparticles of diameter ≈30 nm, Glaco, Soft99) for superhydrophobicity.^30^ The roughness of the nanoscale coatings is ≈50 nm from the atomic force microscopy (AFM) image (Figure 1F). Droplet microfluidics technology enables the fabrication of microcavities and microneedles with the same dimension, thereby allowing us to isolate the effects of topography on hot‐water repellency.

The two surface topographies differ from each other in the stability of Cassie state, as quantified by the breakthrough pressure *P*_break_. The force balance analysis^31^ gives *P*_break_ *S*_unit_(1 − *f*_s_) = γ_up_ *L*_unit_, in which *S*_unit_ is the projected area of a unit cell, *f*_s_ is the solid fraction, γ is the surface tension of water with the subscript “up” representing the upward component, and *L*_unit_ is the length of three‐phase contact line per unit cell. We calculated the breakthrough pressure for hexagonal microcavity (*P*_c_) and microneedle (*P*_n_) surfaces as follows (see Section S1 and Figure S2 in the Supporting Information for the details of the derivation)

(1) $$\{\begin{array}{c} P_{c}=\frac{4\sqrt{3}}{3}\frac{\gamma }{L\sqrt{1-f_{s}}}\max\left(\sin\theta_{E},1\right)\\ P_{n}=-2\sqrt{\frac{4\pi }{3\sqrt{3}}}\frac{\gamma \sqrt{f_{s}}}{L(1-f_{s})}\min(\cos\theta_{E},0) \end{array},\;\mathrm{with}\;f_{s}\leq \frac{\pi }{3\sqrt{3}}$$

in which θ_E_ is the equilibrium contact angle and *L* is the side length of the hexagonal unit cell (Figure 1A,C).

We show that in most situations, *P*_c_ is larger than *P*_n_. First, *P*_n_ = 0 < *P*_c_ when θ_E_ ≤ 90° (*P*_n_ = 0, indicating a fully wetted state of the surface). We then plot *P*_c_ and *P*_n_ versus *f*_s_ ($0<f_{s}<\pi /3\sqrt{3}$) for different θ_E_ (120°, 150°, and 180°) and *L* = 66 µm (the value for microcavities in Figure 1A and microneedles in Figure 1C), as shown in Figure 1G. Unlike *P*_n_, Equation (1) suggests that *P*_c_ is invariant with θ_E_ provided that θ_E_ > 90°, less sensitive to variations in the solid fraction *f*_s_, and converges to a nonzero positive value when *f*_s_ → 0 (Figure 1G). At a given value of θ_E_, *P*_c_ > *P*_n_ when *f*_s_ is smaller than a certain threshold *f*_sc_ (Figure 1G), arising from the re‐entrant and interconnected microstructures of the microcavity surfaces, which leads to larger γ_up_ and *L*_unit_. By equating *P*_c_ and *P*_n_, we find the critical *f*_sc_

(2) $$f_{\mathrm{sc}}=\frac{\sqrt{3}}{\sqrt{3}+\pi \min^{2}\left(\cos\theta_{E},0\right)}$$

depending solely on θ_E_ (Figure 1H). For $f_{s}<f_{\mathrm{sc}}{|}_{\min}=\sqrt{3}/(\pi +\sqrt{3})\approx 0.355$, *P*_c_ is always larger than *P*_n_ at any θ_E_. This result is of practical interest because *f*_s_ values are normally smaller than 0.1 for the purpose of high contact angles and low hysteresis. Therefore, the continuous microcavity topography would endow surfaces with more robust repellency than the discrete microneedle (pillar‐based) topography.

The temperature of water droplets (*T*_d_) affects the breakthrough pressure via influencing θ_E_ and γ. Here, we measure θ_E_ as the contact angle of droplets on nanoparticle coatings, which is larger than 90° for *T*_0_ ≤ *T*_d_ ≤ 90 °C (room temperature *T*_0_ = 23 °C, see Figure 1I). To compare *P*_c_ with *P*_n_, we derive the ratio of *P*_c_ to *P*_n_ when θ_E_ > 90° (the ratio goes into infinity when θ_E_ ≤ 90° because of *P*_n_ = 0)

(3) $$\frac{P_{c}}{P_{n}}=\frac{-1}{\cos\theta_{E}}\sqrt{\frac{\sqrt{3}\left(1-f_{s}\right)}{\pi f_{s}}}$$

As θ_E_ decreases with an increase in *T*_d_, the ratio *P*_c_/*P*_n_ becomes larger at the higher water temperature *T*_d_. Therefore, the hotter the water droplet is, the more robust the repellency becomes on microcavity surfaces than on microneedle surfaces. As Equation (3) diverges when θ_E_ → 90° and *f*_s_ → 0, we expect orders of magnitude enhancement in the stability of the Cassie state by microcavities (Figure S3, Supporting Information); for example, at *f*_s_ = 0.05 and *T*_d_ = 90 °C (θ_E_ = 99.7°), *P*_c_/*P*_n_ = 19.2. Therefore, we expect that the continuous microcavity surface would surpass the discrete microneedle surface in super‐repellency to hot water.

To compare the repellency of the microcavity and microneedle structures, we first investigated the static contact angles of hot‐water droplets on two groups of microcavity and microneedle surfaces with *L* = 80 µm and *L* = 25 µm, respectively (**Figure** **2**A–D). To isolate the effect of surface topography, we fabricated a micropillar surface with the same dimension (*L* = 80 µm and height *H* = 80 µm, see Figure 2E and Figure S4, Supporting Information) as that of the microcavity surface in Figure 2B and compared their repellency to hot‐water droplets. The contact angles θ* and θ_s_ are measured after hot‐water droplets cool down to ensure complete condensation. At room temperature (*T*_0_ = 23 °C), all surfaces are superhydrophobic with [θ* = 164.5°, θ_s_ = 2.3°], [θ* = 153.0°, θ_s_ = 2.5°], [θ* = 167.4°, θ_s_ = 1.9°], [θ* = 161.6°, θ_s_ = 2.3°], and [θ* = 155.4°, θ_s_ = 5°] for structures shown in Figure 2A–E, respectively. Compared to the microcavity surfaces, the higher θ* and lower θ_s_ values of the microneedle surfaces are attributed to their lower solid fraction *f*_s_ and shorter three‐phase contact line. As the droplet temperature *T*_d_ increases, θ* decreases dramatically on the microneedles and micropillars and is eventually smaller than that on the microcavities for both *L* values (Figure 2F). Moreover, the microcavity surfaces display lower θ_s_ values than the microneedle and micropillar surfaces with the same *L* (Figure 2G) for hot droplets. These observations suggest that microcavities outperform the microneedles and micropillars in repelling hot water.

**Figure 2:**
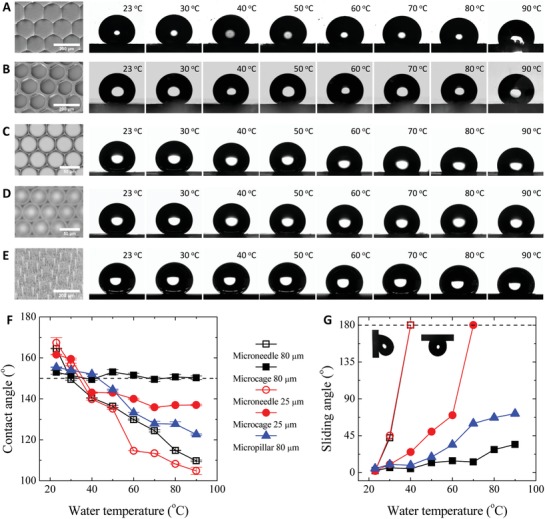
Liquid repellency to static hot‐water droplets. A–E) Contact angles of water droplets with different temperatures on the microneedle surface with *L* = 80 µm (A), microcavity surface with *L* = 80 µm (B), microneedle surface with *L* = 25 µm (C), microcavity surface with *L* = 25 µm (D), and micropillar surface with *L* = 80 µm and height *H* = 80 µm (E), where the dimension is the same as that of the microcavity surface in (B). The left first panel in (A–E) shows the SEM image of the microstructure. F,G) Apparent contact angles (F) and sliding angles (G) of hot‐water droplets on surfaces in (A–E).

The dimension of the microstructure influences hot‐water repellency. Providing larger voids for vapor condensation, larger (*L* = 80 µm) and higher (*H* = 80 µm) structures display higher θ* and lower θ_s_ values than smaller ones (*L* = 25 µm) for hot water (Figure 2F,G). Among all tested surfaces, only the microcavity and micropillar surfaces with *L* = 80 µm and *H* = 80 µm are slippery at all temperatures (up to 90 °C), while the other surfaces become too sticky to allow droplets to roll down when the water temperature is above a certain threshold (Figure 2G, 40 °C for the two microneedles and 70 °C for microcavities with *L* = 25 µm). For structures with the same dimension (*L* = 80 µm and *H* = 80 µm), the microcavity surface outperforms the micropillar surface by displaying a nearly constant θ* (≈150°) and a smaller θ_s_, as the Cassie state of hot‐water droplets are more stable with a higher breakthrough pressure on the microcavity surface than on the micropillar surface, as shown in Equation (3).

The microscopic condensation explains the observed difference in hot‐water repellency on different surfaces. A hot water droplet sitting atop the cold surface evaporates, which provides vapor for the condensation of microdroplets at the lower voids between microstructures. Microdroplets grow in their sizes (with a characteristic timescale *t*_c_ for growing into a size comparable to that of microstructures) until the upper hot‐water droplet cools down to room temperature by evaporation (with a characteristic timescale *t*_e_). The growing microdroplets may have a chance to form liquid bridges that connect the top hot‐water droplet when *t*_e_ > *t*_c_ in the wetting state (**Figure** **3**A) or evaporate to disappear before contacting the top water droplet when *t*_e_ < *t*_c_ in the nonwetting state (Figure 3B).

**Figure 3:**
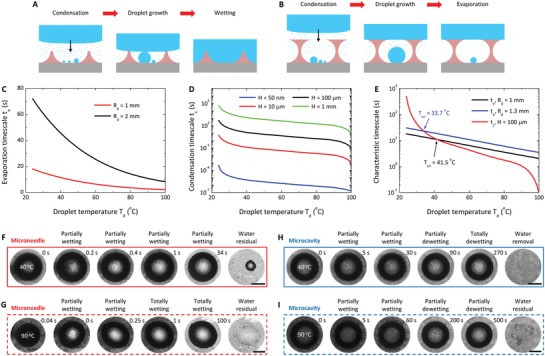
Mechanism accounting for the wetting discrepancy between the microneedle and microcavity surfaces. A,B) Schematics of the wetting processes when a hot‐water droplet is gently deposited onto the microneedle (A) and microcavity (B) surfaces. Compared with microneedles, microcavities tend to remain in their nonwetting state due to microdroplet evaporation. C) Evaporation timescale *t*_e_ for millimeter‐sized droplets. D) Condensation timescale *t*_c_ for structures with different heights *H*. E) Comparison between *t*_e_ and *t*_c_ for millimeter‐sized droplets and *H* = 100 µm. F,G) Snapshots of depositing 40 °C (F) and 90 °C (G) water droplets on a microneedle surface. Increasingly more unit cells are wetted by vapor condensation with time. Finally, the surface is partially wetted by the 40 °C droplet and totally wetted by the 90 °C droplet. H,I) Snapshots of depositing 40 °C (H) and 90 °C (I) water droplets on a microcavity surface. At first, the microcavities are partially wetted by vapor condensation. Then, evaporation of microdroplets induces the dewetting of the surface. Scale bars, 1 mm in (F–I).

The evaporation time scales as *t*_e_ ∼ (*T*_0_ − *T*_d_)/(d*T*/d*t*), where d*T*/d*t* is the rate of temperature change in the evaporating hot droplet. If heat transfer between the droplet and the air by convection and conduction is neglected, then the energy balance equation takes the following form

(4) $$\rho_{d}V_{d}C_{p}\frac{dT}{dt}=-\dot{m}\Delta H_{\mathrm{vap}}$$

where *T*, ρ_d_, *V*_d_, and *C*_p_ are the temperature, density, volume, and constant pressure heat capacity of the droplet, respectively, $\dot{m}$ is the evaporation rate, and Δ*H*_vap_ is the enthalpy of vaporization. The evaporation rate can be derived from vapor diffusion^32^

(5) $$\dot{m}=4\pi R_{d}D_{v}\rho_{a}\frac{P_{\mathrm{sd}}-P_{\mathrm{s0}}}{P_{0}}\frac{M_{v}}{M_{a}}$$

indicating *R*_d_ as the droplet radius, *D*_v_ as the diffusion coefficient of water vapor in air, ρ_a_ as the density of air, *P*_0_ as the atmospheric pressure, *P*_sd_ and *P*_s0_ as the saturation vapor pressure at the temperature of the droplet surface (*T*_d_) and of the air (*T*_0_), respectively, and *M*_v_ and *M*_a_ as the molecular weight of water vapor and of air, respectively. With the assumption of *V*_d_ = 4*πR*_d_^3^/3, the timescale *t*_e_ is then expressed as

(6) $$t_{e}\sim \frac{1}{3}\frac{R_{d}^{2}}{D_{v}}\frac{\rho_{d}C_{p}\left(T_{d}-T_{0}\right)}{\rho_{a}\Delta H_{\mathrm{vap}}}\frac{P_{0}}{P_{\mathrm{sd}}-P_{\mathrm{s0}}}\frac{M_{a}}{M_{v}}$$

Using Equation (6), we calculate *t*_e_ ∼ *O*(10) s for the evaporative cooling of millimeter‐sized water droplets (Figure 3C) when *T*_d_ ranges from *T*_0_ to 100 °C and all values of material properties are chosen at *T*_d_ (Section S2 and Figure S5 in the Supporting Information).

The condensation timescale *t*_c_ can be defined as *t*_c_ ∼ *H*/(d*r*/d*t*), where *H* is the height of microstructures (or equivalently the height of voids between microstructures) and d*r*/d*t* is the growth rate of microdroplets with radius *r*. Because the saturation timescale, *t*_d_ ∼ *L* ^2^/*D*_v_ ∼ *O*(10^−4^) s, is much shorter than *t*_e_ ∼ *O*(10) s, we can assume saturated vapor in microvoids during droplet cooling. The heat transfer rate through a microdroplet (*q*_d_) compensates for the enthalpy change rate of newly condensed vapor, leading to the following formula

(7) $$q_{d}=\rho_{d}\Delta H_{\mathrm{vap}}\frac{dV}{dt}$$

where d*V*/d*t* = 2*πr* ^2^(1 − cos θ_E_)d*r*/d*t* is the volume growth rate of microdroplets. To estimate *q*_d_, we adopt a heat transfer model that accounts for the total thermal resistance arising from the liquid–vapor interface, droplet conduction, and curvature of the microdroplet^33^, ^34^, ^35^

(8) $$q_{d}=\frac{\pi r^{2}(T_{\mathrm{sat}}-T_{s})\left(1-\frac{r_{\min}}{r}\right)}{\frac{1}{2h_{i}(1-\cos\theta_{E})}+\frac{r\theta_{E}}{4k_{d}\sin\theta_{E}}}$$

where *r*_min_ = 2*γT*_sat_/ρ_d_Δ*H*_vap_(*T*_sat_ − *T*_s_), *T*_sat_ = (*T*_d_ + *T*_s_)/2 is the vapor saturation temperature, *T*_s_ is the temperature of solid structures with the assumption of *T*_s_ = *T*_0_ for simplicity, *k*_d_ is the thermal conductivity of the microdroplet, and *h*_i_ is the interfacial heat transfer coefficient. Combining Equations (7) and (8) and choosing *r* = *H*, we have

(9) $$t_{c}\sim \frac{H^{2}\rho_{d}\Delta H_{\mathrm{vap}}}{\left(T_{\mathrm{sat}}-T_{0})(H-r_{\min}\right)}\left(\frac{1}{h_{i}}+\frac{H\theta_{E}(1-\cos\theta_{E})}{2k_{d}\sin\theta_{E}}\right)$$

which ranges from *O*(10^3^) s to *O*(10^−1^) s when *T*_d_ increases from *T*_0_ to 100 °C for *H* = 100 µm, as shown in Figure 3D (see Section S2 in the Supporting Information for materials properties). Since *t*_c_ increases with *H* (Figure 3D), a smaller structure will have a shorter characteristic condensation time, on which wetting is easier to achieve by satisfying the condition of *t*_c_ < *t*_e_. Therefore, surfaces with smaller structures are more susceptible to wetting by hot‐water droplets than those with larger structures, consistent with our observations in Figure 2.

Figure 3E contrasts *t*_e_ with *t*_c_ for *H* = 100 µm and millimeter‐sized water droplets, similar to the case (Figure 2A,B, *L* = 80 µm) we investigate in the present study (see Figure S6 in the Supporting Information for the comparison with other *H* values). The value of *t*_e_ is comparable with *t*_c_ in the transitional temperature range of 30 °C < *T*_d_ < 40 °C (depending on the droplet size), below which *t*_e_ < *t*_c_ indicates nonwetting and above which *t*_e_ > *t*_c_ suggests wetting. This prediction agrees well with our observations for the microneedle surfaces, on which hot droplets firmly stick due to full wetting when *T*_d_ ≥ 40 °C (Figure 2G). For microcavity surfaces, the presence of the top continuous structures may provide physical barriers for preventing the coalescence between the hot droplets and microdroplets, thereby retaining the nonwetting state even when *T*_d_ ≥ 40 °C. We observed partial wetting (Figure 3F, some of the voids between the microneedles are wetted) and total wetting (Figure 3G, all voids underneath the hot‐water droplet are wetted) of microneedle surfaces by 40 °C and 90 °C droplets, respectively. In contrast, dewetting due to the evaporation of microdroplets takes place on microcavities (fully dewetting at 40 °C in Figure 3H and partially dewetting at 90 °C in Figure 3I). After the removal of water droplets, large water residuals are left on the microneedles, while no (40 °C) or only a few (90 °C) residuals are left on microcavities, highlighting the significant difference in the wetting state between the two surface topographies.

When a hot‐water droplet impinges a superhydrophobic surface, there is a chance that the droplet bounces off the surface without wetting if the condensation microdroplets are not large enough to form liquid bridges during the contact period of the hot droplet with the solid surface. We observed the bouncing of hot‐water droplets on both microneedles (**Figure** **4**A) and microcavities (Figure 4B). On the microneedle surface, only droplets with *T*_d_ ≤ 50 °C rebound fully from the surface, but those with higher temperature (*T*_d_ ≥ 60 °C) rebound partially, leaving behind some water residuals sticking on the surface due to wetting (the size of the sticking residual increases as *T*_d_ increases). In comparison, all droplets with *T*_0_ ≤ *T*_d_ ≤ 90 °C can bounce off the microcavity surface fully without wetting (Figure 4A,B and Figure S7, Supporting Information).

**Figure 4:**
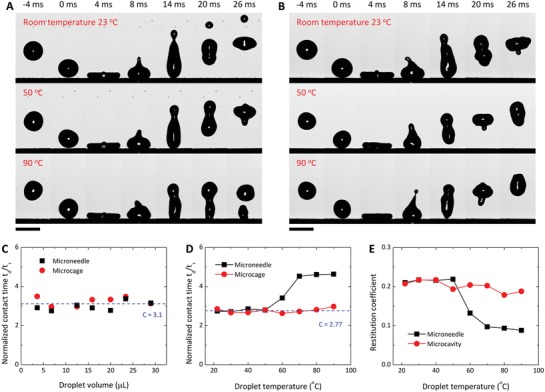
Liquid repellency to impinging hot‐water droplets. A,B) Snapshots of 23, 50, and 90 °C droplets impinging on the microneedle (A) and microcavity (B) surfaces. Hot‐water droplets can completely bounce off the microcavity surface at all temperatures but fail in doing so on the microneedle surface when the droplet temperature is larger than 50 °C. C) Droplet contact time (*t*_d_) normalized by the inertia‐capillary timescale (*t*_τ_) for impingement of room‐temperature droplets. D,E) Time ratio *t*_d_/*t*_τ_ (D) and restitution coefficient ε (E) for impingement of droplets with different temperatures. The *t*_d_/*t*_τ_ and ε remain invariant for the microcavity surface but start to increase and decrease for the microneedle surface when the droplet temperature is larger than 50 °C, respectively. Scale bars, 3 mm in (A,B).

The contact time *t*_d_ of a bouncing droplet scales as the inertia‐capillary timescale, *t*_τ_ = (ρ_d_ *R*_d_^3^/γ)^1/2^

(10) $$t_{d}=C\sqrt{\frac{\rho_{d}R_{d}^{3}}{\gamma }}$$

where *C* is the prefactor.^36^ Fitting of experimental data gives *C* = 3.1 for room‐temperature droplets bouncing off the microneedle and microcavity surfaces when the Weber number We ≈ 19 where We = 2ρ_d_ *U* ^2^ *R*_d_/γ with U being the velocity of the droplet at impact (Figure 4C). For a millimeter‐sized water droplet with *T*_0_ ≤ *T*_d_ ≤ 90 °C, *t*_b_ is on the order of *O*(10^−2^) s (Figure 4A,B and Figure S8). We find that the prefactor *C* is constant (*C* = 2.77) for hot‐water droplets impinging on the microcavity surface but increases when *T*_d_ ≥ 60 °C on the microneedle surface (Figure 4D), consistent with the bouncing dynamics documented in Figure 4A,B. The restitution coefficient, defined as ε = *H*_r_/*H*_0_ (*H*_r_ and *H*_0_ represent the maximum height of the rebounding droplets and the initial release height of droplets, respectively), is used to quantify the loss of droplet momentum after bouncing. The value of ε basically remains constant for the microcavity surface but undergoes a sharp decrease at *T*_d_ = 60 °C on the microneedle surface (Figure 4E) as a result of surface wetting.

For hot impinging droplets, the competition between *t*_d_ and *t*_c_ determines the wetting state, i.e., *t*_d_ > *t*_c_ for wetting, whereas *t*_d_ < *t*_c_ for nonwetting. Experiments indicate that *t*_d_ ∼ *O*(10^−2^) s (see Figure 4A,B), much smaller than *t*_c_ ∼ *O*(10^3^–10^−1^) s (Figure 3E, *H* = 100 µm) for microneedles and microcavities, which predicts the not‐wetting of both surfaces during droplet impingement. Nevertheless, the prediction is violated by the impingement of droplets with *T*_d_ ≥ 60 °C on the microneedle surface. Microscopic observation reveals quite fast wetting (within 4 ms) on the microneedle surface for 70 °C droplets (**Figure** **5**A); in contrast, no wetting was observed on the microcavity surface under the same conditions (Figure 5B). As such, a train of hot‐water droplets sticks on an inclined microneedle surface (Figure 5C), whereas each droplet can shed away the inclined microcavity surface (Figure 5D).

**Figure 5:**
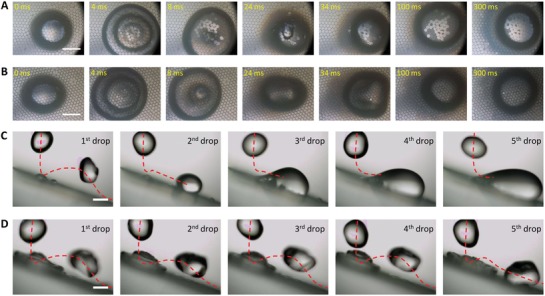
Wetting dynamics for the impingement of hot‐water droplets. A,B) Snapshots of a 70 °C droplet impinging on the microneedle (A) and microcavity (B) surfaces. The microneedle surface is rapidly wetted when contacting the droplet, whereas the microcavity surface is barely wetted during the whole process. C,D) Superimposed images showing the impingement of a train of 70 °C droplets on the tilted microneedle (C) and microcavity (D) surfaces. Droplets stick on the microneedle surface but readily shed away from the microcavity surface. Scale bars, 1 mm in (A,B) and 2 mm in (C,D).

The ultrafast wetting of the microneedle surface is attributed to its low breakthrough pressure. When a droplet impinges on a solid surface, an effective water hammer pressure (*P*_ewh_ ∼ ρ_d_ *Uc*, with *c* being the velocity of sound in water) and a dynamic pressure (*P*_d_ = ρ_d_ *U* ^2^/2) compete with the breakthrough pressure for the wetting transition during the initial contact and latter spreading stages, respectively.^37^, ^38^ The lower breakthrough pressure of microneedles makes them more prone to wetting than microcavities. In this case, the condensation time is estimated to be *t*_c_ ∼ *H*_eff_/(d*r*/d*t*) for the microneedle surface, with the effective height *H*_eff_ ranging between 100 µm and 50 nm (the roughness of nanoparticle coatings, see Figure 1F). Using *H*_eff_, *t*_c_ is reduced by several orders of magnitude (exemplified by *H* = 50 nm, Figure 3D), *t*_c_ < *O*(10^−3^) s < *t*_d_ ∼ *O*(10^−2^) s, which predicts the wetting on the microneedle surface.

The difference in the repellency to hot‐water droplets on microneedle and microcavity surfaces renders the heat transfer process distinct from each other. Compared to the microneedle surface, the heat flux from the hot‐water droplet to the glass substrate (*q*_ls_″) is expected to be lower on the microcavity surface because of the entrapped air that performs as a thermal insulation layer with a larger thermal resistance (**Figure** **6**A). Therefore, the microcavity‐coated substrate would have a smaller temperature (*T*_bott_), as observed in Figure 6B. Upon the deposit of hot‐water droplets, *T*_bott_ undergoes a sharp rise and peaks within several seconds, followed by a gradual decrease to a level lower than the room temperature *T*_0_ (inset in Figure 6B), which is attributed to droplet evaporation.

**Figure 6:**
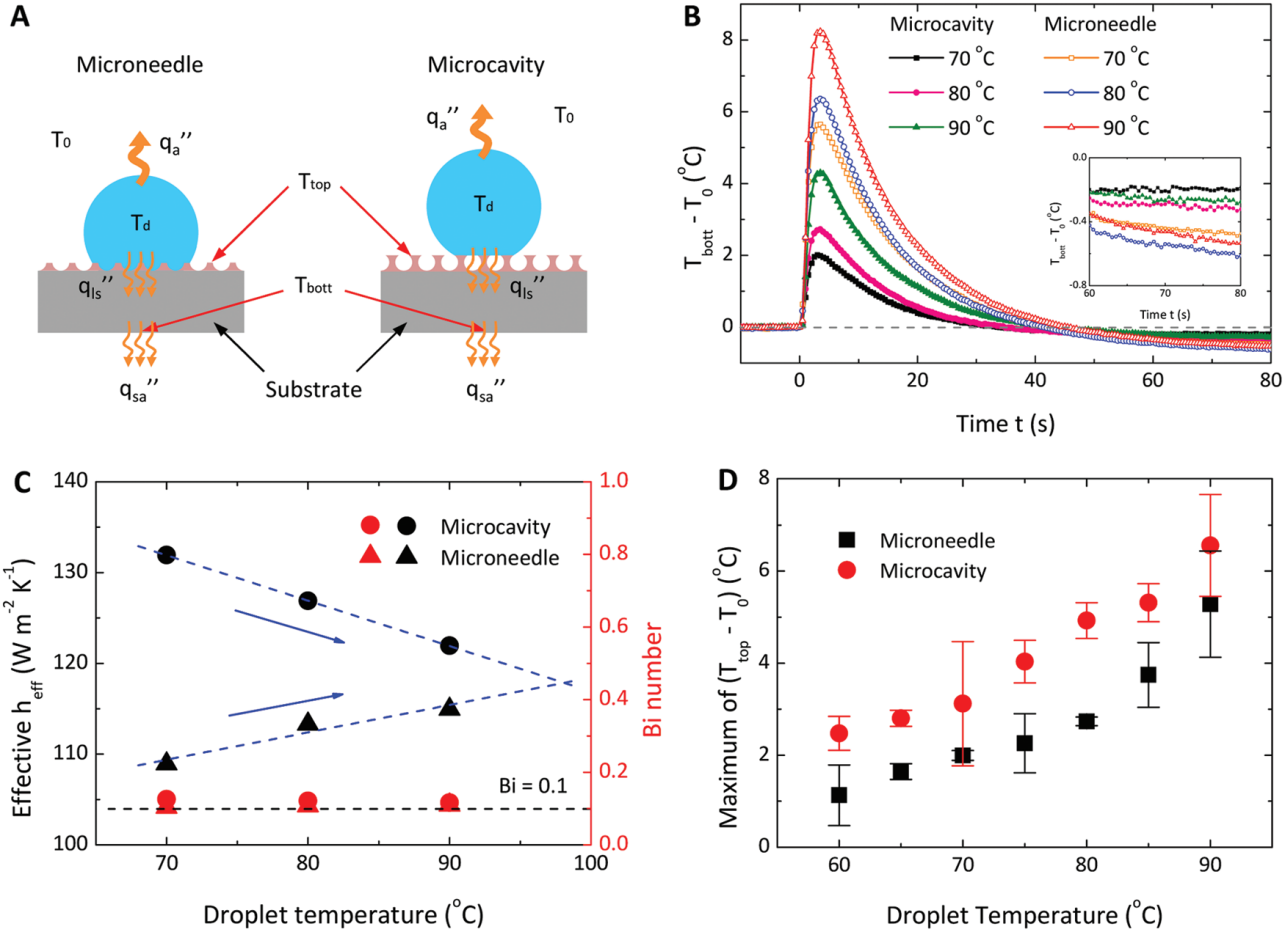
Wetting‐dependent heat transfer. A) Schematics of the heat transfer on the microneedle (left) and microcavity (right) surfaces. The hot droplet with temperature *T*_d_ releases heat into the surrounding air (temperature *T*_0_) at a flux of *q*_a_″ and through the surface into the underneath substrate at a flux of *q*_ls_″. There also exists a heat flux (*q*_sa_″) from the heated substrate to the surrounding air. Depending on the wetting states, the heat flux *q*_ls_″ would be different for the two surfaces, which finally induces differences in the temperature of the substrate (*T*_bott_) and the top surface (*T*_top_) for the two cases. B) Evolution of the substrate temperature (*T*_bott_) after hot‐water droplets are deposited onto the microneedle and microcavity surfaces. C) Effective heat transfer coefficient *h*_eff_ and Biot number (Bi) for substrates underneath the two surfaces. D) Variation of the maximum temperature of the top surface (*T*_top_) with the droplet temperature. *T*_top_ is measured at a position 3 mm away from the center of the hot droplets.

We adopted a lumped capacitance model^39^ to describe the transient heat transfer of substrate cooling

(11) $$\frac{T_{\mathrm{bott}}(t)-T_{\min}}{T_{\max}-T_{\min}}=\exp\left\{-\frac{h_{\mathrm{eff}}}{\rho_{s}z_{s}C_{s}}t\right\}$$

where *T*_min_ and *T*_max_ are the minima and maxima of the substrate temperature, respectively, *h*_eff_ is the effective heat transfer coefficient, and ρ_s_, *z*_s_, and *C*_s_ are the density, height, and heat capacity of the substrate, respectively. We obtained the exponent of (−*h*_eff_/ρ_s_ *z*_s_ *C*_s_) by fitting the experimental data (Figure S9, Supporting Information) and calculated *h*_eff_ with known values of ρ_s_, *z*_s_, and *C*_s_, as shown in Figure 6C. Using *h*_eff_, we determined the Biot number Bi = *h*_eff_ *z*_s_/*k*_s_ ≈ 0.1 (Figure 6C) with *k*_s_ being the thermal conductivity of glass, indicating that it is reasonable to assume a uniform temperature distribution within the glass substrate in the lumped capacitance model.

The *h*_eff_ reflects the cooling rate of the substrate; a larger *h*_eff_ represents a faster cooling down rate. The cooling rate is determined by and increases with the heat flux difference (*q*_sa_″ − *q*_ls_″), where *q*_sa_″ is the heat flux from the substrate to the air. As *q*_ls_″ is lower through microcavities than through microneedles due to air‐layer insulation, we conjecture that *h*_eff_ would be larger for the substrate coated with the microcavity surface than with the microneedle surface, which agrees well with experiments (Figure 6C). When the droplet temperature is higher, more vapor condensation takes place in microcavities such that the insulation of the air layer becomes weaker, reducing the difference in *h*_eff_ between the substrates coated by the two surfaces. From linear fitting, the *h*_eff_ of the two surfaces intersects at ≈*T*_d_ = 98.1 °C, where the air layer vanishes and the microcavity surface is fully wetted by condensation, behaving exactly the same as the microneedle surface. The intersection method may be useful in predicting the critical droplet temperature at which a surface entirely loses the liquid repellency.

We further investigated the temperature at the top of the two surfaces, *T*_top_ (Figure 6A). Compared to discrete microneedles, continuous microcavities are more favorable to heat diffusing radially outward away from the center of the hot droplets along the top surface due to the interconnectivity of the solid structures. The enhanced lateral thermal diffusion explains the observed higher maxima of *T*_top_ on the microcavity surface than on the microneedle surface (Figure 6D).

As one potential application, we performed exothermic reactions using neutralization between HCl and NaOH as a model system on the two surface topographies. Litmus was added into the HCl aqueous droplet to visualize the reaction (**Figure** **7**A,B). The contact angle of the HCl droplet decreases slightly by 5.7° (from 137.8° to 132.1°, Figure 7B) after the addition of NaOH for the reaction on the microcavity surface, in stark contrast to the large decrease of 23.1° (from 133.1° to 111.8°, Figure 7A) on the microneedle surface. Once neutralization begins, numerous bubbles appear at the bottom of microneedles and then slowly disappear (Figure 7C). In contrast, bubbles only occur at the top layer of microcavities with much smaller sizes (too small to be visible by naked eyes without magnification, Figure 7D). The microbubbles arise from the expansion of nanoscale voids (Figure 1F) heated by the exothermic reaction. The collapse of bubbles may induce surface wetting that accounts for the decreased contact angles after the reaction. Compared to the microneedle surface, wetting is less detrimental to the microcavity surface because it is limited to the top layer. We observed the sliding down of droplets along the tilted microcavity surface after the reaction between 4 m HCl and 4 m NaOH, indicating the slippery state.

**Figure 7:**
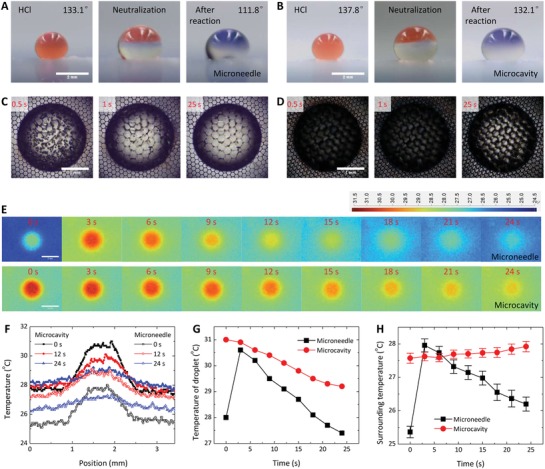
Neutralization of the droplets. A,B) Optical images showing the neutralization between a 4.2 µL 6 m HCl droplet and a 5.8 µL 6 m NaOH droplet on the microneedle (A) and microcavity (B) surfaces. Litmus is added into the HCl droplet for better visualization when the color changes from red into blue as the pH increases. C,D) Snapshots of the neutralization after the coalescence between 5 µL 6 m HCl and 5 µL 6 m NaOH droplets on the microneedle (C) and microcavity (D) surfaces. As neutralization releases heat, nanoscale voids expand into microbubbles at the bottom of the microneedles and the top of the microcavities during the reaction, followed by a fading away due to temperature decrease. This process can induce the wetting of microneedles and microcavities. E) Infrared images showing the temperature field during the neutralization reaction on the microneedle (upper row) and microcavity (lower row) surfaces. F) Spatial distribution of temperature across the center of reacting droplets at different times. G) Variation in the maximum temperature of reacting droplets with time. H) Variation in the average surrounding temperature with time. Scale bars, 2 mm in (A,B,E) and 1 mm in (C,D).

Thermography allowed us to reveal the difference in the evolution of the temperature field after the reaction on the two surfaces (Figure 7E). The droplet is a heat source with a higher temperature than the surroundings (Figure 7E,F). Compared with the droplet on the microneedle surface, the droplet on the microcavity surface is always higher in temperature and cools down at a lower rate (Figure 7G). Moreover, the surrounding temperature gradually increases for the microcavity surface, while it quickly decreases after an initial increase for the microneedle surface (Figure 7H). Such differences are highly related to the wetting state of the surface. The observations are consistent with the heat transfer analysis in Figure 6. The nonwetting of the microcavity surface provides a thermal insulating layer of air between the droplet and the substrate, by which the droplet cools down more slowly and the microcavity surface is gradually heated due to the lateral thermal diffusion. However, the wetting of the microneedle surface transfers most of the heat from the droplet to the substrate, which induces a more rapid cooling down of the droplet and the surroundings.

Indeed, the distinct heat transfer characteristics of the super‐repellent microcavity surface would be very useful in the application of self‐cleaning and protective coatings to prevent the coated objects from simultaneous fouling and thermal damage. For example, the surface can be used in digital microfluidics for performing exothermic reactions and manipulating droplets with varied temperatures in chemical and biological assays; it can also be used in the fabrication of protective clothing for thermal isolation when hot water contacts clothes.

In summary, we identify that the continuous microcavity surface displays more robust super‐repellency to hot water than the discrete microneedle surface. The former distinguishes from the latter by the way in which the breakthrough pressure depends on the solid fraction and water temperature. Our theoretical model shows that a smaller solid fraction and higher water temperature leads to more robustness in the stability of liquid repellency on microcavity surfaces. Topographically, the interconnected solid structures provide physical barriers that segregate the microcavity surface into top and bottom layers, on which the dynamics of wetting and heat transfer are almost independent of each other. Therefore, most wetting events are confined to the top layer that is in direct contact with the hot water, while the bottom layer is nonwetted to maintain super‐repellency for the microcavity surface. In contrast, the microneedle structures are less robust in shielding the bottom surface from hot water due to the lack of physical barriers and are thereby more susceptible to wetting.

We utilized droplet microfluidics to fabricate microcavities and microneedles with the same dimension, both of which are coated with silanized silica nanoparticles for superhydrophobicity at room temperature. The microcavity surface displays super‐repellency to water droplets at elevated temperatures, exhibiting higher apparent contact angles and lower adhesion than the microneedle surface; dynamically, hot‐water droplets totally bounce off the microcavity surface at all tested temperatures (up to 90 °C), whereas sticking droplets only partially rebound from the microneedle surface when the temperature of the water droplets is higher than 50 °C. We developed theoretical models to estimate the characteristic timescales for droplet evaporation, vapor condensation, and droplet bouncing, which elucidate the observed differences in wetting dynamics between the two surface topographies. The hot‐water super‐repellency endows the microcavity surface with a distinct heat transfer process where the entrapped air cushion imposes a thermal insulating layer between the hot droplet and substrate. The microcavity surface retains the nonwetting state after performing the neutralization reaction. We expect that the microcavity topography will open new avenues for applications of super‐repellency coupled with heat transfer. For example, microcavity surfaces can be used as coatings for self‐cleaning and thermal insulation.

## Experimental Section

*Fabrication of Microcavity, Microneedle, and Micropillar Surfaces*: The fabrication of microcavity surfaces was described in detail in the previous studies^23^, ^25^, ^28^ and involves four stages: emulsion generation, emulsion deposition, solvent evaporation, and template removal. First, monodisperse emulsions are produced in a capillary microfluidic device, where silicone oil (20 cSt; Aldrich) droplets (with 2 wt% Dow Corning 749 Fluid as the surfactant) were dispersed in a poly(vinyl alcohol) (PVA, Mw 13 000–23 000, 87–89% hydrolyzed; Aldrich) aqueous solution. Then the emulsion was deposited onto a glass substrate, after which the silicone oil droplets self‐assembled into ordered hexagonal arrays while the PVA solution evaporates at room temperature.^28^ The evaporation of water solidifies the PVA membrane, which wraps the silicone oil droplet arrays. Finally, the PVA membrane was immersed in toluene (99.8%; Sigma‐Aldrich) for 1 h to wash out the silicone oil droplets, followed by drying the membrane in a vented hood. Eliminating the silicone oil droplets produces microcavities of the same size. To prevent the PVA surface from dissolving into water, PVA was crosslinked with glutaraldehyde (50 wt% in water; Sigma‐Aldrich). A detailed description of tuning the type and dimension of microstructures is included in the previous study.^28^

The microneedle surface was fabricated by peeling off the top layer of the microcavity surface using adhesive tape. This ensures the fabrication of microcavity and microneedle surfaces with the same dimension for comparison.

The height *H* of the microfluidically fabricated microcage structure is related to its unit length *L* in the form of *H* = *πL*/3 ≈ *L*, as reported in the previous work.^28^ Silicon micropillar arrays were fabricated with *L* = 80 µm and *H* = 80 µm by etching. First, a layer of aluminum was deposited on the silicon wafer by e‐beam evaporation (TF500, HHV), on top of which a layer of photoresist (AZ MIR 701, MicroChemicals) was coated and then exposed with direct laser writing (DWL 66+, Heidelberg Instruments). After developing, the exposed aluminum was removed by an inductively coupled plasma (ICP, GSE200, NAURA) with Cl_2_ and BCl_3_. Finally, silicon was etched by the ICP with O_2_ and SF_6_ for the production of micropillars with predesigned heights.

To obtain superhydrophobicity at room temperature, all surfaces were coated with silanized silica nanoparticles (diameter ≈30 nm) using commercialized Glaco (Soft99). After coating, the surfaces were baked at 80 °C for 30 min.

*Visualizing the Wetting Dynamics*: The macroscopic wetting dynamics of water droplets were visualized by a high‐speed camera (Phantom M110) equipped with a camera lens (Sigma, 30 mm, f/1.4). The microscopic visualization of droplet wetting was performed using an inverted optical microscope (Nikon Eclipse TS100) equipped with a high‐speed camera (Phantom M110). Recorded images and videos were analyzed using ImageJ software for the measurement of contact angles and sliding angles. In characterizing the wetting by static hot‐water droplets, the images/videos were taken ≈1 min after the deposition of droplets to allow them to cool down. 10 µL water droplets were used for the measurement of contact angles.

*Neutralization Reaction*: To perform neutralization, an aqueous HCl droplet was first deposited on the test surface, followed by dripping an aqueous NaOH droplet. The coalescence of the two droplets initiated the neutralization reaction. The concentration of the acid and alkali solutions ranged from 2 to 6 m. To visualize the reaction, litmus was added into the HCl solution, which displays a red color; an increase in the pH value with the addition of NaOH for the reaction can change the color from red to blue. To enhance the visual contrast, the reaction between a 4.2 µL 6 m HCl droplet and a 5.8 µL 6 m NaOH droplet was used in Figure 7A,B. For other experiments, 5 µL HCl and 5 µL NaOH droplets were used, such as those in Figure 7C–H. Neutralization was also performed using 2 and 4 m solutions, where the droplet rolls off both the microcavity and microneedle surfaces after the reaction between 2 m HCl and 2 m NaOH solutions and rolls off only the microcavity surface after the reaction between 4 m HCl and 4 m NaOH solutions.

*Characterization*: The surface morphology was characterized by scanning electron microscopy (SEM, Hitachi S4800 and S3400N and Zeiss EVO MA10) and atomic force microscopy (Bruker MultiMode 8). The temperature of the glass substrate (*T*_bott_) and test surfaces (*T*_top_) was automatically measured by thermocouples (*K*‐type) using a data acquisition system (Keysight 34970A Data Acquisition/Switch Unit). The temperature field of the surfaces was measured by an infrared thermal imager (Fluke, TiS40) for the neutralization experiment.

## Conflict of Interest

The authors declare no conflict of interest.

## Supporting information

Supplementary
