# Supplementary material for: Topography‐Directed Hot‐Water Super‐Repellent Surfaces
Source: Adv Sci (Weinh). 2019 Jul 30;6(18):1900798. doi: 10.1002/advs.201900798 (PMC6755536; doi:10.1002/advs.201900798)
Supplement: Supplementary file 1 — Supplementary [file ADVS-6-1900798-s001.pdf]

Copyright WILEY-VCH Verlag GmbH & Co. KGaA, 69469 Weinheim, Germany, 2019.

ADVANCED SCIENCE
Open Access

Supporting Information

for *Adv. Sci.,* DOI: 10.1002/advs.201900798

Topography-Directed Hot-Water Super-Repellent Surfaces

*Pingan Zhu, Rifei Chen, and Liqiu Wang**

Supporting information for

Topography-directed hot-water super-repellent surfaces

Pingan Zhu[1,2], Rifei Chen[1,3], Liqiu Wang[1,2,*]

[1] *Department of Mechanical Engineering, the University of Hong Kong, Hong Kong, China*
[2] *HKU-Zhejiang Institute of Research and Innovation (HKU-ZIRI), Hangzhou 311300, Zhejiang, China*
[3] *Department of Materials Science and Engineering, Southern University of Science and Technology, Shenzhen 518055, China*
*Corresponding author: lqwang@hku.hk

Section S1. Breakthrough pressure for microcavity and microneedle surfaces

The breakthrough pressure can be estimated by a force balance analysis between the pressure inside the liquid and the capillary force [1]: $P_{\mathrm{break}} S_{\mathrm{int}} = \gamma_{\mathrm{up}} L_{\mathrm{unit}}$ where S_{int} is the interfacial area with $S_{\mathrm{int}} = S_{\mathrm{unit}}(1 - f_{\mathrm{s}})$ indicating f_{s} as the solid fraction of the surface, γ_{up} is the upward component of the capillary force with $\gamma_{\mathrm{up}} = \gamma \max\left(\sin(\theta_{\mathrm{E}} - \varphi_{\mathrm{min}}), 1\right)$ for all $\theta_{\mathrm{E}} > \varphi_{\mathrm{min}}$ and $L_{\mathrm{tot}} = 0$ for $\theta_{\mathrm{E}} \le \varphi_{\mathrm{min}}$, θ_{E} is the equilibrium contact angle, φ_{min} is the minimum geometric angle, S_{unit} is the area of a unit cell, and L_{unit} is the length of the three-phase contact line per unit cell.

For hexagonal-shaped microcavities with side-length of L (Figure S2A), $S_{\mathrm{unit}} = 3\sqrt{3}L^2/2$, $L_{\mathrm{unit}} = 6L'$ with L' being the opening size of the microcavity on the top layer, $1 - f_s = \left(L'/L\right)^2$, and $\varphi_{\mathrm{min}} = 0°$ [2, 3]. Therefore, the breakthrough pressure for hexagonal microcavity surface (P_{c}) is

$$P_{\mathrm{c}} = \frac{4\sqrt{3}}{3} \frac{\gamma}{L\sqrt{1 - f_{\mathrm{s}}}} \max(\sin\theta_{\mathrm{E}}, 1). \tag{S1}$$

For hexagonally-arrayed microneedles or micropillars with needle spacing of L (Figure S2B), $S_{\mathrm{unit}} = 3\sqrt{3}L^2/2$, $L_{\mathrm{unit}} = 4\pi R_{\mathrm{n}}$ where R_{n} is the radius of the pillar, $f_s = 2\pi R_{\mathrm{n}}^2 / S_{\mathrm{unit}}$, and $\varphi_{\mathrm{min}} = 90°$. As such, the breakthrough pressure for the microneedle surface (P_{n}) is

$$P_n = -2\sqrt{\frac{4\pi}{3\sqrt{3}}}\frac{\gamma\sqrt{f_s}}{L(1-f_s)}\min(\cos\theta_E, 0). \quad (S2)$$

For microcavity surfaces, f_s can be as large as 1, but for microneedle surfaces, the maximized f_s takes place when $R_n = L/2$, which gives $f_s \le \frac{2\pi(L/2)^2}{3\sqrt{3}L^2/2} = \frac{\pi}{3\sqrt{3}}$.

Section S2. Materials properties used in predicting t_e and t_c

Material properties are adopted from Ref. [4] and *The Engineering ToolBox* [5]. Polynomial fitting gives the following estimate in materials properties for calculation of t_e and t_c.

(1) Constant pressure heat capacity of water:

$C_p(T)$ = 746.991818156573 − 7.805817792999217 · T + 0.03591031468381318 · T^2 − 0.00007534188033874774 · T^3 + 6.002331002086895 · 10^{-8} · T^4 (J/mol K). (S3)

(2) Enthalpy of vaporization for water:

$\Delta H_{vap}(T)$ = 140.089135866380 4 − 1.1075090469397693 · T+ 0.0060671484125004805 · T^2 − 0.000017965644709547645 · T^3 + 2.701443164501017 · 10^{-8} · T^4 − 1.639328808335716 7 · 10^{-11} · T^5 (kJ/mol). (S4)

(3) Density of water:

$\rho_d(T)$ = 199154.4601714155 − 4798.0756418478795 · T + 49.29021474331967 · T^2 − 0.2789427537511922 · T^3 + 0.000940307948317107 · T^4 − 1.8896524752273574 · 10^{-6} · T^5 + 2.097335403903993 · 10^{-9} · T^6 − 9.922135928718443 · 10^{-13} · T^7 (kg/m^3). (S5)

(4) Density of air:

$\rho_a(T)$ = 6.9493 − 0.05541 · T + 0.000229362 · T^2 – 5.20832 · 10^{-7} · T^3 + 6.16538 · 10^{-10}· T^4 − 2.97967 · 10^{-13} · T^5 (kg/m^3). (S6)

(5) Diffusion coefficient of water vapor in air:

$D_v(T) = 0.000170689 - 1.62917 \cdot 10^{-6} \cdot T + 5.87484 \cdot 10^{-9} \cdot T^2 - 8.10761 \cdot 10^{-12} \cdot T^3 + 4.14185 \cdot 10^{-15} \cdot T^4$ (m²/s). (S7)

(6) Saturation vapor pressure:

$P_{sd}(T) = 3962550 - 46276.69121 \cdot T + 171.29142 \cdot T^2 - 0.08611 \cdot T^3 - 0.000731868 \cdot T^4 + 1.13617 \cdot 10^{-6} \cdot T^5$ (Pa). (S8)

(7) Saturation vapor pressure at T_0: $P_{s0} = 2785.756$ (Pa).

(8) Atmospheric pressure: $P_0 = 101325$ (Pa).

(9) Molecular weight of air: $M_a = 28.6$ (g/mol).

(10) Molecular weight of water vapor: $M_v = 18$ (g/mol).

To calculate the condensation timescale t_c, the interfacial heat transfer coefficient h_i is expressed as [6-8], $h_i = \frac{2\sigma}{2-\sigma}\frac{\rho_v \Delta H_{vap}^2}{T_{sat}}\left(\frac{M_v}{2\pi T_{sat} R}\right)^{1/2}$, where ρ_v is the vapor density, σ is the accommodation coefficient which we assume here to be $\sigma = 0.8$, and R is the universal gas constant with $R = 8.3144598$ J/mol K.

(11) Thermal conductivity of water:

$k_d(T) = -6.67086 + 0.0774 \cdot T - 3.17362 \cdot 10^{-4} \cdot T^2 + 5.96648 \cdot 10^{-7} \cdot T^3 - 4.30358 \cdot 10^{-10} \cdot T^4$ (W/m K). (S9)

(12) Vapor density:

$\rho_v(T) = 15.24844 - 0.24086 \cdot T + 0.00141 \cdot T^2 - 3.62499 \cdot 10^{-6} \cdot T^3 + 3.48562 \cdot 10^{-9} \cdot T^4$ (kg/m³). (S10)

(13) The surface tension of water [9]:

$\gamma(T) = 75.714 - 0.1414 \cdot T - 2.5399 \cdot 10^{-4} \cdot T^2$ (mN/m). (S11)

(14) Equilibrium contact angle (from Figure 1I)

$\theta_E(T) = 8.14017 + 28.77589 \cdot T - 1.89206 \cdot T^2 + 0.05684 \cdot T^3 - 8.77534 \cdot 10^{-4} \cdot T^4 + 6.76608 \cdot 10^{-6} \cdot T^5 - 2.06754 \cdot 10^{-8} \cdot T^6$ (°). (S12)

Materials properties (1)-(6) and (11)-(12) are fitted as a function of the absolute temperature (with unit K), and those of (13)-(14) are as a function of the Celsius temperature (with unit °C). We plot materials properties (1)-(6) and (11)-(13) in Figure S5. The equilibrium contact angle is shown in Figure 1I in the context. The comparison between t_e and t_c for varies microstructure height H and droplet radius R_d is shown in Figure S6.

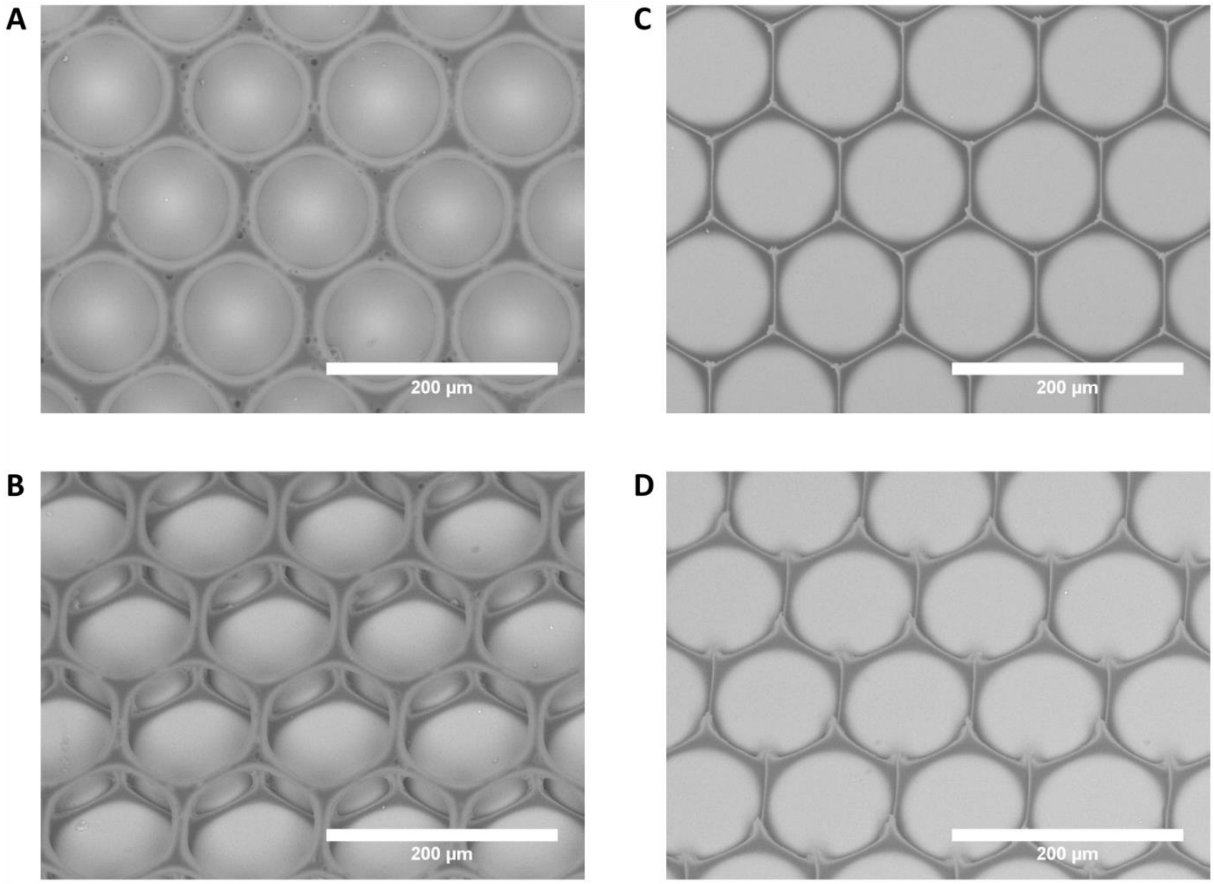

Figure S1. SEM images of the bare microcavity (A-B) and microneedle (C-D) surfaces in top view (A and C) and 30° tilted view (B and D) before coating of hydrophobic silica nanoparticles.

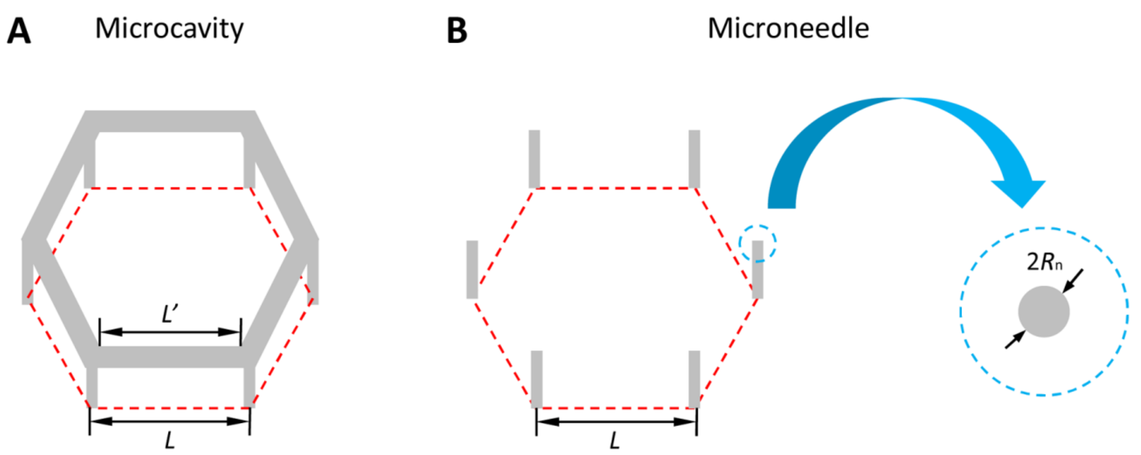

Figure S2. Schematics of the hexagonal unit cell of the microcavity (A) and microneedle (B) surfaces.

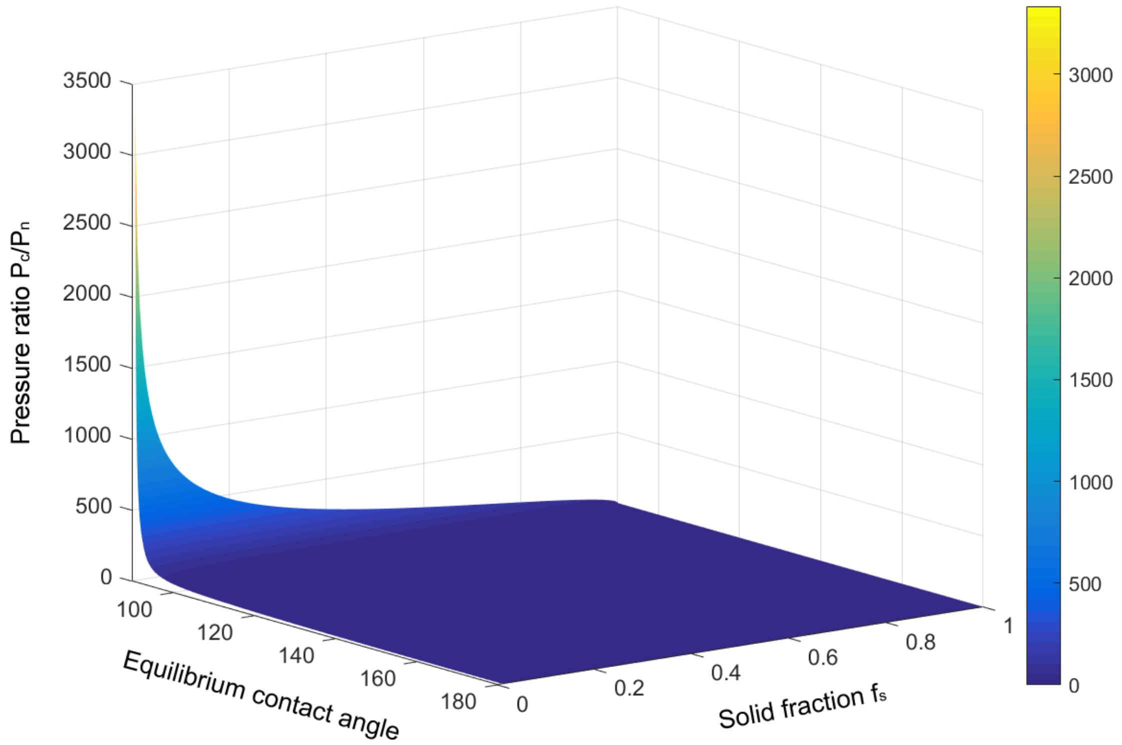

Figure S3. The ratio of the breakthrough pressure P_c/P_n as a function of the equilibrium contact angle θ_E and solid fraction f_s. P_c/P_n diverges when either θ_E approaches 90° or f_s approaches 0. Note that when $\theta_E < 90°$, $P_n = 0$, and the ratio P_c/P_n goes into infinity.

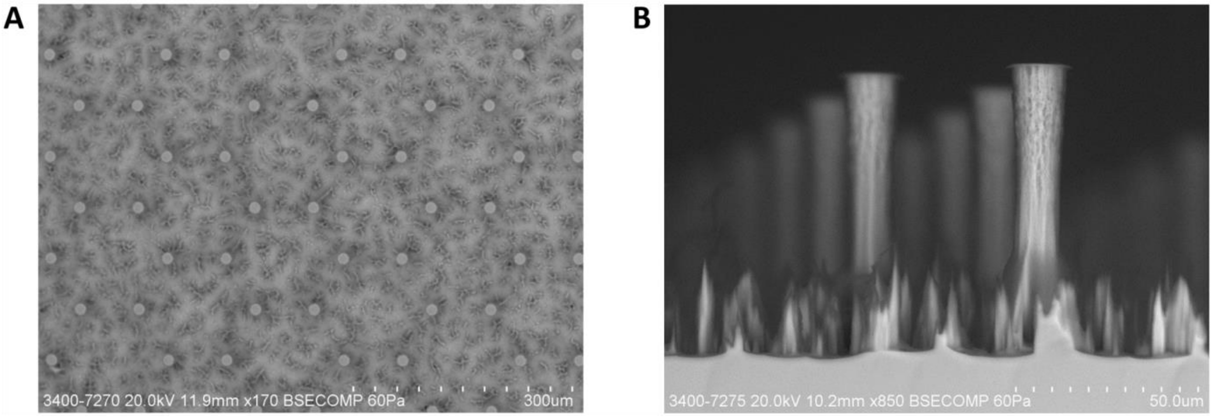

Figure S4. Micropillar surface. (A) Top view of micropillar hexagonal arrays with a unit length of L = 80 μm. (B) Side view of micropillar with a height of H = 80 μm.

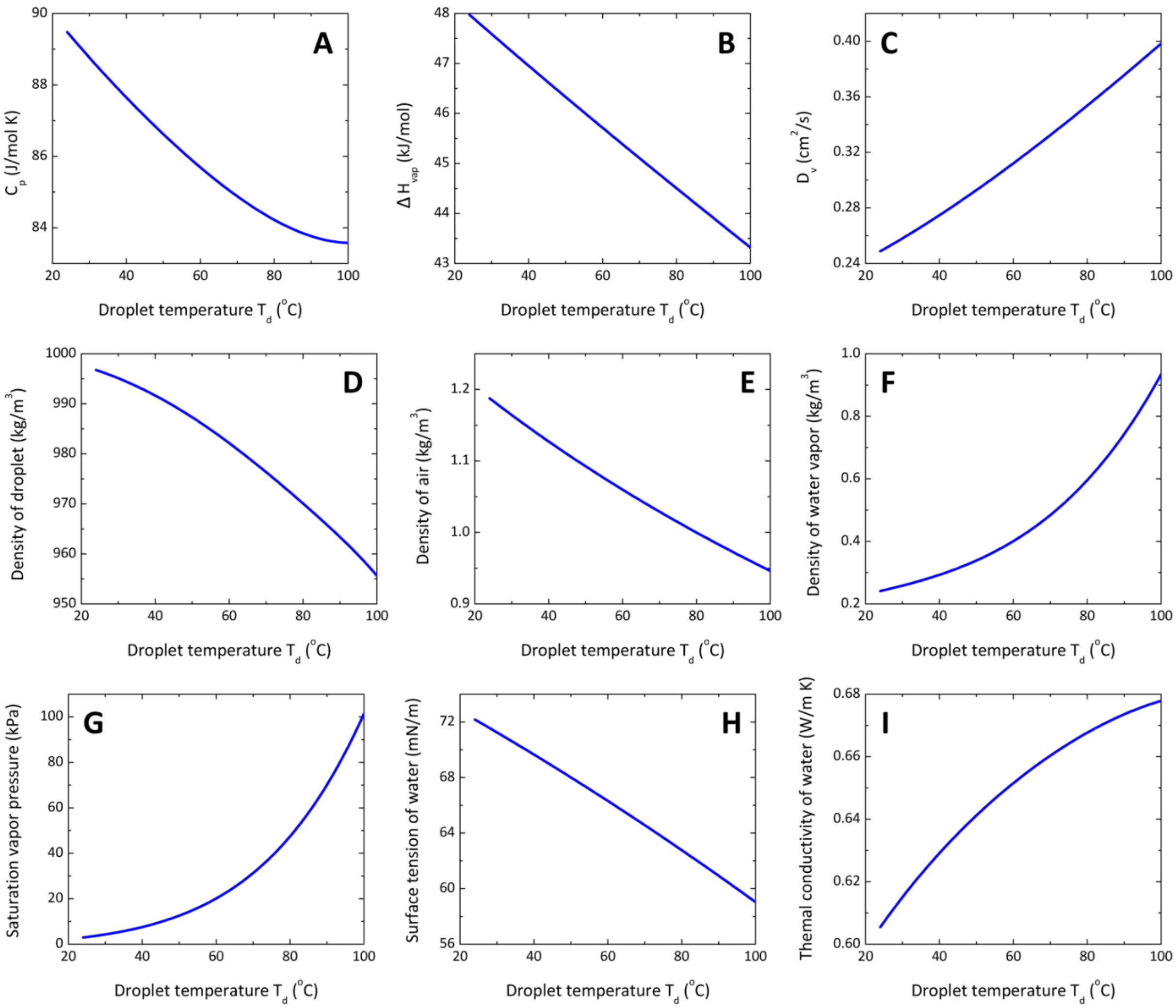

Figure S5. Polynomial fittings of materials properties. (A) Constant pressure heat capacity of water, C_p. (B) Enthalpy of vaporization for water, ΔH_{vap}. (C) Diffusion coefficient of water vapor in air, D_v. (D) Density of water droplet, ρ_d. (E) Density of air, ρ_a. (F) Density of water vapor, ρ_v. (G) Saturation water vapor pressure, P_{sd}. (H) Surface tension of water, γ. (I) Thermal conductivity of water, k_d.

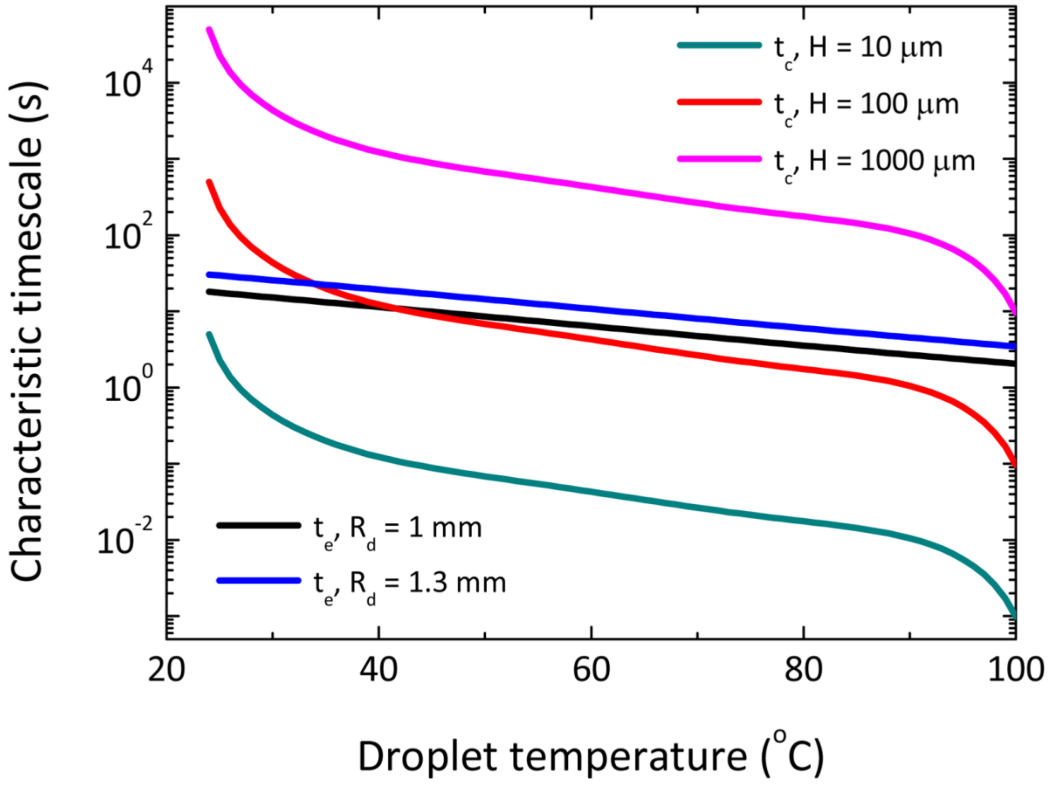

Figure S6. Comparison between evaporation time t_e and condensation time t_c. t_e is calculated for two droplet radius R_d, 1 mm (4.2 μL) and 1.3 mm (9.2 μL), and t_c is calculated for three microstructure height H, 10 μm, 100 μm, and 1 mm. Microstructures with H = 100 μm are of high interest to observe the transition from non-wetting to wetting states (where t_e intersects with t_c), larger structures with H = 1 mm lie in the non-wetting regime ($t_e \sim O(10)$ s $< O(10^5\text{-}10)$ s $\sim t_c$) whereas smaller structures with H = 10 μm are in the wetting regime ($t_e \sim O(10)$ s $> O(10\text{-}10^{-3})$ s $\sim t_c$).

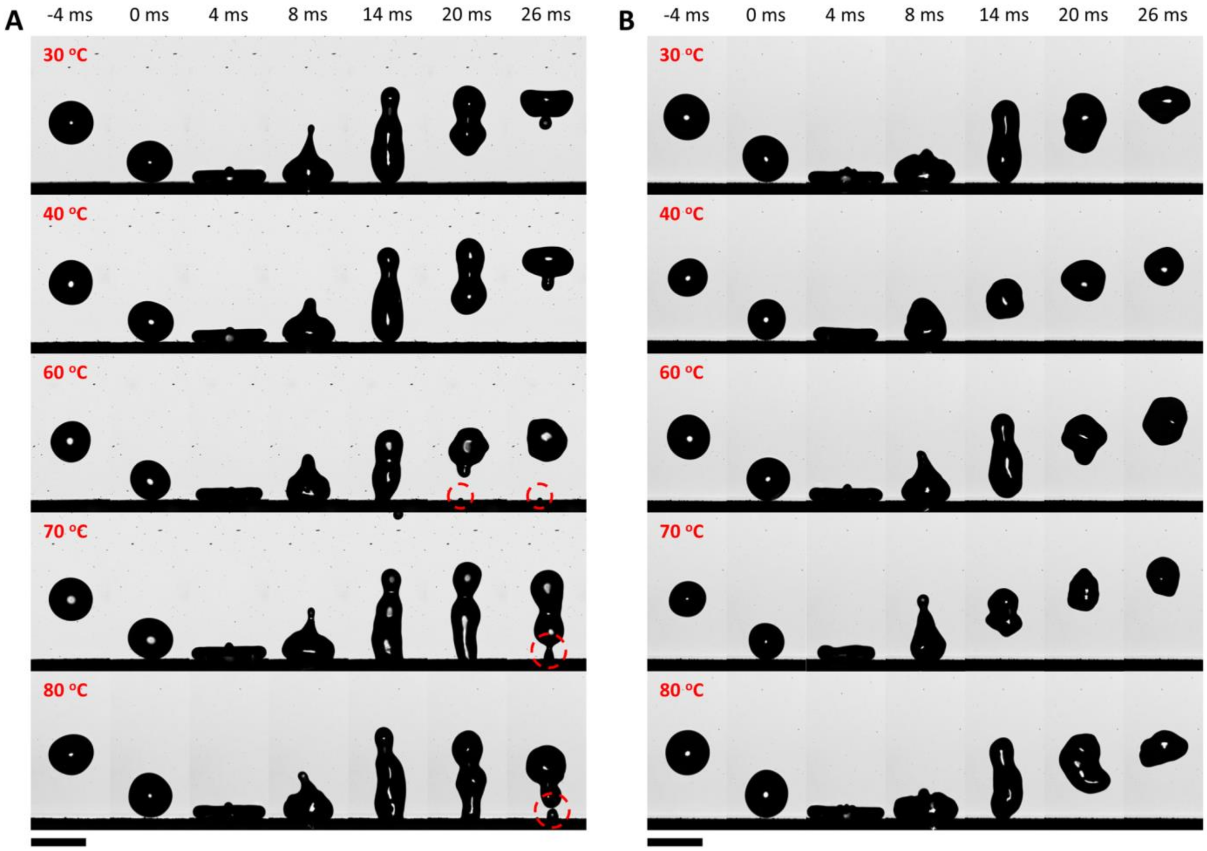

Figure S7. Snapshots of hot-water droplets impinging on the microneedle (A) and microcavity (B) surface. Droplets with the temperature of 30 °C, 40 °C, 60 °C, 70 °C, and 80 °C are displayed, supplementary to Figures 3A-3B in the context for droplets with the temperature of 23 °C, 50 °C, and 90 °C. Hot-water droplets can completely bounce off the microcavity surface at all temperatures but fail in doing so on the microneedle surface when droplet temperature is 60 °C and larger, where sticking liquid residuals are left behind, as highlighted by the dashed circles in (A).

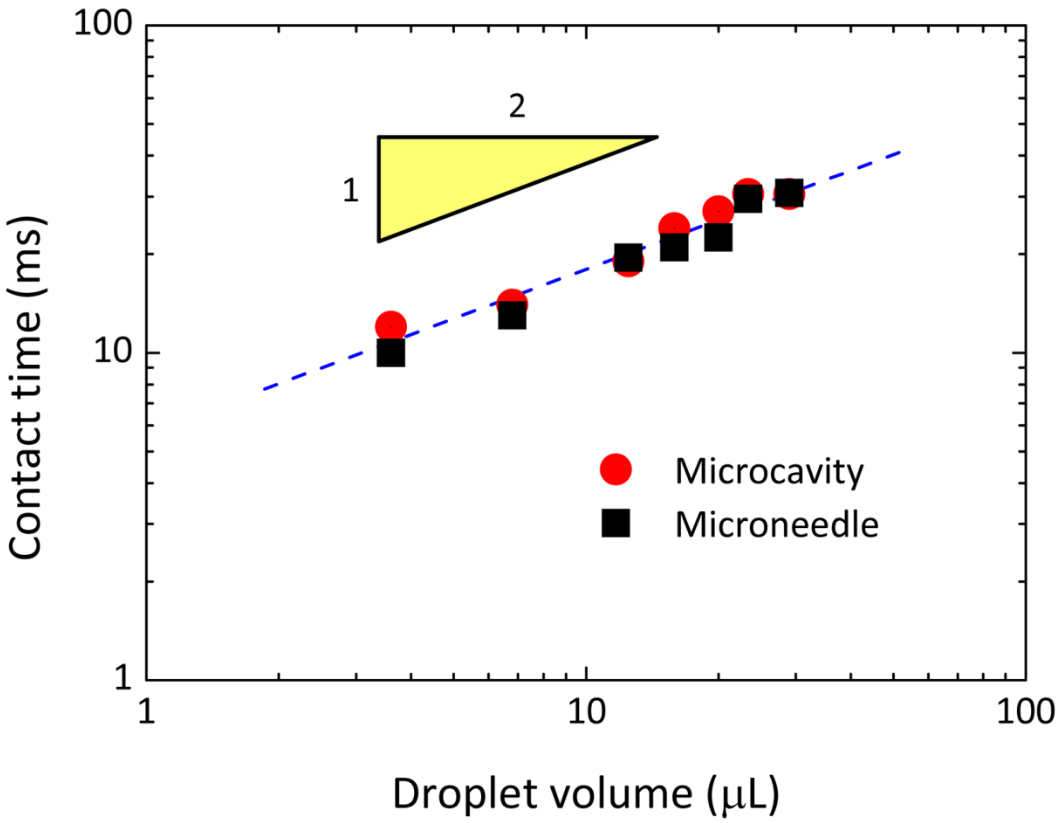

Figure S8. Variation of the contact time t_d with the droplet volume V_d at room temperature. t_d scales as V_d in the form of $t_d \sim V_d^{1/2}$ for both microcavity and microneedle surfaces, consistent with the result that t_d is proportional to the inertia-capillary time $t_\tau = (\rho_d R_d^3/\gamma)^{1/2}$ with a constant prefactor factor C. t_d is on the order of $O(10)$ ms for tested droplets.

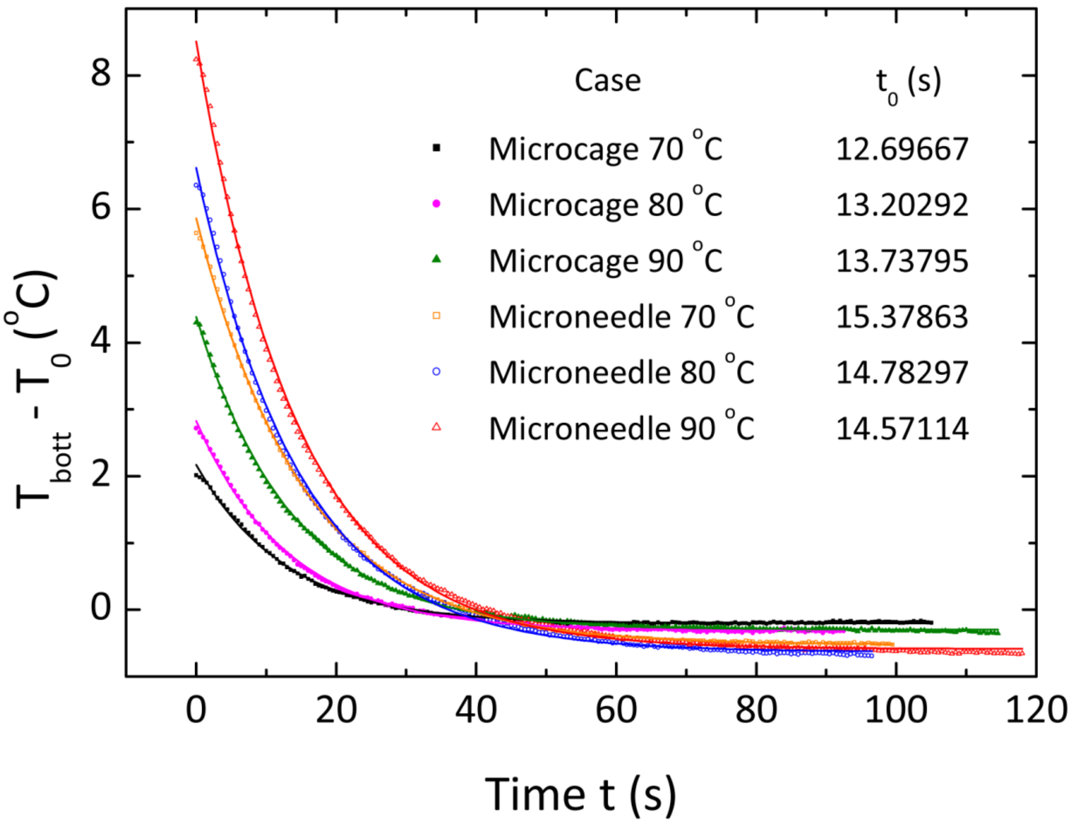

Figure S9. Exponential curve fitting of experimental results during cooling. The fitting function is in the form of $(T_{bott} - T_0) = a*\exp(-t/t_0) + b$, where t_0 equals $\rho_s z_s C_s/h_{eff}$, indicating the characteristic cooling time for each case. Smaller t_0 implies faster cooling down.

References

[1] A. Tuteja, W. Choi, J. M. Mabry, G. H. McKinley and R. E. Cohen, *Proc. Natl. Acad. Sci. U.S.A.*, **2008**, *105*, 18200.

[2] P. A. Zhu, T. T. Kong, Y. Tian, X. Tang, X. W. Tian and L. Q. Wang, *Mater. Horiz.*, **2018**, *5*, 1156.

[3] P. A. Zhu, T. T. Kong, X. Tang and L. Q. Wang, *Nat. Commun.*, **2017**, *8*, 15823.

[4] D. Schlesinger, J. A. Sellberg, A. Nilsson and L. G. Pettersson, *J. Chem. Phys.* , **2016**, *144*.

[5] DOI: The Engineering ToolBox. See https://www.engineeringtoolbox.com/.

[6] K. Rykaczewski, *Langmuir*, **2012**, *28*, 7720.

[7] S. Shiri, A. Murrizi and J. Bird, *Micromachines*, **2018**, *9*, 566.

[8] I. Tanasawa, in *Advances in Heat Transfer*, eds. J. P. Hartnett, T. F. Irvine and Y. I. Cho, Elsevier, 1991, vol. 21, pp. 55-139.

[9] G. Loglio, A. Ficalbi and R. Cini, *J. Colloid Interface Sci.*, **1978**, *64*, 198.
